# Digitalisierung, Daten und KI in Medizin und Pflege. Virtuelles Nachwuchskolloquium des „Netzwerks Junge Medizinethik“ (JMED): Online, 09.02.–15.06.2021

**DOI:** 10.1007/s00481-021-00650-1

**Published:** 2021-07-28

**Authors:** Philipp Karschuck, Svenja Wiertz, Frank Ursin, Wenke Liedtke, Kris Vera Hartmann, Florian Funer

**Affiliations:** 1 Klinik und Poliklinik für Urologie, Medizinische Fakultät Carl Gustav Carus, TU Dresden, Fetscherstr. 74, 01307 Dresden, Deutschland; 2 Institut für Ethik und Geschichte der Medizin, Universität Freiburg, Freiburg, Deutschland; 3 Institut für Geschichte, Theorie und Ethik der Medizin, Universität Ulm, Ulm, Deutschland; 4 Fachbereich Soziale Arbeit, Evangelische Hochschule Rheinland-Westfalen-Lippe, Bochum, Deutschland; 5 Institut für Geschichte und Ethik der Medizin, Ruprecht-Karls-Universität Heidelberg, Heidelberg, Deutschland; 6 Lehrstuhl für Moraltheologie/Theologische Ethik, Eberhard Karls Universität Tübingen, Tübingen, Deutschland

## Entstehung des Nachwuchskolloquiums

Seit 2018 bietet das „Netzwerk Junge Medizinethik“ (JMED) ein Forum für Nachwuchswissenschaftler:innen in der Akademie für Ethik in der Medizin e. V. (AEM). Das Netzwerk steht jungen Medizinethiker:innen wie PostDocs und fortgeschrittenen Doktorand:innen offen, die im Bereich der Medizinethik in Forschung, Lehre oder Beratung tätig sind. JMED möchte den fachlichen und methodischen Austausch seiner Mitglieder stärken sowie zur Weiterqualifikation und Vernetzung beitragen. Für diesen Zweck werden regelmäßig Workshops und Netzwerktreffen organisiert. Seit 2021 bietet JMED zudem eine Plattform für digitales Peer Mentoring in der Medizinethik (PRIME) an.^1^

Im Zuge der COVID-19-Pandemie entfielen die etablierten Formate für den wissenschaftlichen Nachwuchs. Gerade diese Gruppe profitiert von Kolloquien hinsichtlich der Vertiefung laufender Forschungsaktivitäten sowie zur Entwicklung neuer Ideen und Ansätze für künftige Projekte. Darüber hinaus bieten diese Formate die Möglichkeit zur eigenen Profilbildung und Qualitätssicherung.

Als pragmatische Alternative haben sich Onlineformate etabliert, wie das virtuelle JMED-Get-Together (jeden zweiten Montag im Monat ab 20:00 Uhr). Auch der dritte JMED-Workshop vom 15. bis 17. Januar 2021 hat sich durch die digitale Umstellung erneut als vielbesuchtes und produktives Ereignis erwiesen – nicht nur, aber auch, weil er zur Entstehung eines neuen Formats beigetragen hat: dem JMED-Nachwuchskolloquium.

Mit dem Ziel, ein Forum für den niederschwelligen Fachaustausch zu aktuellen Projekten von JMED-Mitgliedern und medizinethischen Nachwuchswissenschaftler:innen zu schaffen, fanden zwischen dem 09.02. und 15.06.2021 regelmäßige Online-Treffen via Zoom statt (dienstags, 12 bis 13 Uhr). Dabei standen nach einer kurzen Projektvorstellung (20 min) die ausführliche Diskussion und der Austausch im Vordergrund (40 min). Das Kolloquium startete mit zehn Vorträgen zum Thema „Digitalisierung, Daten und KI in Medizin und Pflege“ (Tab. 1).

**Table 1:** 

Datum	Titel	Referent:in
09.02.2021	Künstliche Intelligenz in der Radiologie: Explicability als fünftes bioethisches Prinzip?	Frank Ursin, Ulm
16.02.2021	Broad Consent als neuer Standard der informierten Einwilligung?	Svenja Wiertz, Freiburg
23.02.2021	Entscheidungshilfe Prostatakrebs: ein Online-Tool als Katalysator für shared decision-making?	Philipp Karschuck, Dresden
13.04.2021	Ethische Aspekte in der Entwicklung von KI-basierten Assistenzsystemen	Kris Vera Hartmann, Heidelberg
20.04.2021	Die Dialektik der Personalisierung. Zur instrumentellen Vernunft in der digitalen Gesundheitsversorgung	Giovanni Rubeis, Heidelberg
27.04.2021	Unterstützungssysteme in der Häuslichkeit: Ethische Analyse sensorbasierter Alarmsysteme	Wenke Liedtke, Bochum
04.05.2021	Autonomie und Vertrauen mit Blick auf Sleep-Tracking Apps	Regina Müller, Tübingen
01.06.2021	Deep Medicine – Deep Empathy? Ärztliche Empathie im Zeitalter Künstlicher Intelligenz	Sarah Albrecht, Münster
08.06.2021	Technologie und empirische Medizinethik? Ansätze einer Forschungsmethode am Beispiel der Corona-Warn-App	Joschka Haltaufderheide, Bochum
15.06.2021	Sag, wie hältst du es mit den Daten? Einblick in die Dokumentationspraxis Sozialer Arbeit	Diana Schneider, Bielefeld

## Querschnittthemen der Diskussion

Im Rahmen der ausführlichen Darstellungen und Diskussionen der einzelnen Projekte ergaben sich Querschnittthemen, die wiederholt aufgegriffen wurden. Diese Themen, so der Eindruck der Autor:innen, bilden jenseits des JMED-Nachwuchskolloquiums aktuell wichtige Fragestellungen der medizinischen Ethik und ihrer Methodik ab. Dabei sind insbesondere die Themen Digitalisierung, der Umgang mit Daten und der Einsatz künstlicher Intelligenz zu nennen. Aufgrund des Werkstattcharakters des Kolloquiums werden im Folgenden primär Fragen formuliert, die gegenwärtig aus unserer Sicht einer weiteren Vertiefung bedürfen.

### Künstliche Intelligenz und Digitalisierung in der Medizin: zukünftige Anwendungsfelder und Voraussetzungen einer erfolgreichen Implementierung

Angesichts rasanter Entwicklungen in den Bereichen von Digitalisierung und künstlicher Intelligenz ist die Medizinethik in der interdisziplinären Zusammenarbeit zunehmend aufgefordert, Potenziale, Chancen und Risiken innovativer Ansätze zu bewerten sowie alternative Handlungs- und Entwicklungsstrategien gegeneinander abzuwägen. Gerade in Zeiten der Pandemie rückte die Notwendigkeit zur unmittelbaren ethischen Güterabwägung bei medizinischen Handlungsstrategien und Maßnahmen in den Fokus aktueller Debatten.

Als wichtig wurde in allen Beiträgen innerhalb des Kolloquiums erachtet, dass eine ethische Bewertung anhand konkreter technologischer Artefakte vorgenommen werden sollte. Daher wurden Technologien oder Anwendungen analysiert, die im besten Fall bereits (kommerziell) verfügbar sind. Die besprochenen Beispiele aus laufenden Forschungsprojekten reichten von diagnostischer KI in der Radiologie über eine leitliniengerechte Online-Entscheidungshilfe für Prostatakrebspatienten, komplexe Assistenzsysteme für ältere und physisch eingeschränkte Menschen sowie Apps zur Schlafüberwachung bis hin zur Corona-Warn-App.

Die dabei diskutierten ethischen Fragen kreisten um drei zentrale Aspekte: erstens die Veränderung der Beziehung zwischen Ärzt:innen, Patient:innen und technischen Anwendungen; zweitens die Qualität der Daten und der ihnen inhärenten systematischen Verzerrungen, die sich über das maschinelle Lernen auch in algorithmischen Systemen abbilden; drittens die Spannung zwischen dem Ziel technischer Entwicklungen als Unterstützung des Menschen und dem ökonomisch motivierten Ersatz menschlicher Akteure, beispielsweise als kurzfristige Kompensation eines Versorgungsmangels. Dies führte zur Grundfrage, welche menschlichen Kompetenzen durch technische Anwendungen überhaupt ersetzt werden können oder sollten. Welche Auswirkungen haben Entscheidungshilfen auf das „Shared decision-making“ im Ärzt:innen-Patient:innen-Vertrauensverhältnis? Lässt sich die Versorgungsqualität durch partizipatorische Elemente und innovative, evidenzbasierte Informationsvermittlung steigern? Mit welchen unvorhergesehenen, vielleicht sogar unerwünschten Anwendungen ist zu rechnen? Wie ist ein Recht auf Ablehnung algorithmischer Entscheidungen zu begründen und wie umfangreich müssen Patient:innen über deren Nutzung informiert werden? Ist überhaupt absehbar, ob das Erreichen der anberaumten Ziele technischer Entwicklung wahrscheinlich ist?

### Interdisziplinarität und Methodik: Wer bearbeitet die anstehenden Herausforderungen?

Die Medizinethik versteht sich als interdisziplinäre Wissenschaft. Sie verbindet (moral-)philosophisch begründete, normative Theorieansätze mit qualitativen und quantitativen empirischen Arbeitsweisen. Sofern sie sich mit Kontexten der Technikentwicklung und Digitalisierung auseinandersetzt, werden Kenntnisse und Perspektiven von Informatiker:innen und Ingenieur:innen zunehmend unverzichtbar. Immer wieder stellt sich die Herausforderung, wie der Spagat zwischen diesen Disziplinen (gut) gelingen kann.

Herausforderungen hierbei wurden im Kolloquium etwa auf begrifflicher Ebene diskutiert: Wie gehen wir beispielsweise mit dem unterschiedlichen Gebrauch des Begriffs „Privatheit“ in philosophischen, ingenieurs- und informationswissenschaftlichen Kontexten um, insbesondere dann, wenn mit den unterschiedlichen Bedeutungsdimensionen verschiedene Anliegen verbunden sind? Dabei stellt sich auch die Frage, wie eine „Übersetzung“ der unterschiedlichen Fachsprachen gelingen und ein ernsthafter interdisziplinärer Dialog vorangebracht werden kann.

Eine weitere Frage ergab sich aus der begrenzten Erklärbarkeit algorithmischer Systeme. Inwieweit ist aus ethischer Sicht vertretbar, dass häufig nicht nachvollzogen werden kann, warum die eingesetzte KI die eine oder andere Entscheidung getroffen hat? Ist es für den Bereich der Medizinethik notwendig, die Erklärbarkeit (explicability) eines Outcomes/einer Entscheidung als eigenes (bioethisches) Prinzip zu formulieren?

In der medizinethischen Forschung werden mittlerweile viele ethische Bewertungsrahmen jenseits des von der Europäischen Kommission favorisierten Modells „responsible research and innovation“ (RRI) zur Förderung von Forschungs- und Entwicklungs-Vorhaben genutzt. Es scheint daher gegenwärtig angesichts der Vielzahl angewandter Methoden noch offen, ob sich etwa das MEESTAR- oder CANVAS-Modell, Check-Listen-Methoden oder Werte-Analysen durchsetzen werden. Im Kolloquium wurde beispielhaft diskutiert, ob eine philosophisch orientierte dialektische Methode gemessen am disruptiven Potenzial neuer Technologien zielführender sein könnte.

Ein wichtiger Diskussionspunkt war darüber hinaus, ob technische Artefakte überhaupt Gegenstand ethischer Bewertung sein können. Wie groß ist die Gefahr eines Missverständnisses, wenn wir eine Technik bewerten wollen, diese de facto aber nur im veränderlichen Anwendungskontext als soziale Praxis normativ in den Blick genommen werden kann? Sofern sich die Bewertungen auf menschliche Handlungen beziehen sollen (und nicht eine ethische Handlungsfähigkeit von Technologien unterstellt wird), muss eine entsprechende Bewertung neuer technischer Artefakte mit einbeziehen, welche menschlichen Handlungsmöglichkeiten diese erwartbar hervorbringen könnten.

### Transgressives Potenzial digitaler Gesundheitsanwendungen: Medizinprodukt oder Lifestyle-App?

Angesichts der Vielfalt bereits heute genutzter und zukünftig zu erwartender Technologien fällt die Differenzierung distinkter Anwendungsfelder zunehmend schwer. Zwar richten sich Produkte wie Entscheidungsunterstützungssysteme etwa für Prostatakrebspatient:innen oder in der häuslichen Pflege noch deutlich an medizinisches oder pflegerisches Personal als Nutzer:innen und können der Klassifikation „Medizinprodukt“ zugeordnet werden. Apps für Patient:innen und/oder „gesunde“ Menschen weisen jedoch auf einen fließenden Übergang zwischen „medizinischer Anwendung“ und „Lifestyle-App“ hin. Denn diese digitalen Gesundheitsanwendungen bieten zumeist die Nutzung in beiderlei Weise an. Beispielweise können „Sleep-Tracking-Apps“ aufgrund ihrer Überwachung des persönlichen Schlafrhythmus sowohl den vorhandenen als auch den angestrebten Lebensstil unterstützen, gleichzeitig aber auch durch die Detektierung medizinisch relevanter Vorkommnisse im Rahmen diagnostischer Prozesse wertvoll sein. Aus medizinethischer Perspektive sind hierbei Aufklärung, digitale (Gesundheits‑)Kompetenz und Datensouveränität der Nutzer:innen zentral: Liegt ein Lifestyle-Produkt vor, wird die Anwendung diagnostisch oder therapeutisch genutzt und wie werden die Nutzer:innendaten gesammelt und verarbeitet? Wie stellt sich das Spannungsverhältnis von „digital divide“ und „digital literacy“ im Gesundheitswesen dar?

## Fazit und Ausblick

Das Nachwuchskolloquium hat sich als digitales Format bewährt, auch wenn es den unmittelbaren kollegialen Austausch nicht ersetzen kann. Insbesondere die Gelegenheit zum niederschwelligen und überregionalen Austausch wurde von den Initiator:innen und Teilnehmer:innen in den vergangenen Monaten wertgeschätzt. Wir streben daher an, das Format auch in Zukunft weiterzuführen, und freuen uns auf viele weitere Themenschwerpunkte aus dem Feld der Ethik in der Medizin.

Um die Impulse aus den Diskussionen produktiv für die jeweiligen Projekte und Publikationen wirken zu lassen, macht das JMED-Nachwuchskolloquium mit dem Themenschwerpunkt „Digitalisierung, Daten und KI in Medizin und Pflege“ zunächst eine Pause. Eine Wiederaufnahme des Schwerpunkts und ein Update der vorgestellten Projekte ist für das Frühjahr 2022 geplant.

Im Herbst und Winter 2021 wird das JMED-Nachwuchskolloquium einen anderen Themenschwerpunkt in den Blick nehmen und Fragen zu „Diversität in der Medizin(‑ethik)“ behandeln. Erste spannende Vorträge sind bereits geplant; wir freuen uns auf viele weitere Vorschläge (aktuelle Informationen finden sich auf www.junge-medizinethik.de).

## Footnotes

1 www.junge-medizinethik.de. Zugegriffen: 15.07.2021.

